# Oxpholipin 11D: An Anti-Inflammatory Peptide That Binds Cholesterol and Oxidized Phospholipids

**DOI:** 10.1371/journal.pone.0010181

**Published:** 2010-04-14

**Authors:** Piotr Ruchala, Mohamad Navab, Chun-Ling Jung, Susan Hama-Levy, Ewa D. Micewicz, Hai Luong, Jonathan E. Reyles, Shantanu Sharma, Alan J. Waring, Alan M. Fogelman, Robert I. Lehrer

**Affiliations:** 1 Department of Medicine, David Geffen School of Medicine, University of California Los Angeles, Los Angeles, California, United States of America; 2 Department of Radiation Oncology, David Geffen School of Medicine, University of California Los Angeles, Los Angeles, California, United States of America; 3 Department of Chemistry and Center for Macromolecular Modeling & Materials Design, California State Polytechnic University, Pomona, California, United States of America; University of Helsinki, Finland

## Abstract

**Background:**

Many Gram-positive bacteria produce pore-forming exotoxins that contain a highly conserved, 12-residue domain (ECTGLAWEWWRT) that binds cholesterol. This domain is usually flanked N-terminally by arginine and C-terminally by valine. We used this 14-residue sequence as a template to create a small library of peptides that bind cholesterol and other lipids.

**Methodology/Results:**

Several of these peptides manifested anti-inflammatory properties in a predictive *in vitro* monocyte chemotactic assay, and some also diminished the pro-inflammatory effects of low-density lipoprotein in apoE-deficient mice. The most potent analog, Oxpholipin-11D (OxP-11D), contained D-amino acids exclusively and was identical to the 14-residue design template except that diphenylalanine replaced cysteine-3. In surface plasmon resonance binding studies, OxP-11D bound oxidized (phospho)lipids and sterols in much the same manner as D-4F, a widely studied cardioprotective apoA-I-mimetic peptide with anti-inflammatory properties. In contrast to D-4F, which adopts a stable α-helical structure in solution, the OxP-11D structure was flexible and contained multiple turn-like features.

**Conclusion:**

Given the substantial evidence that oxidized phospholipids are pro-inflammatory *in vivo*, OxP-11D and other Oxpholipins may have therapeutic potential.

## Introduction

Oxysterols and oxidized lipids are important mediators in many signaling pathways [1], [2]. Oxysterols may interact with various molecular targets, including liver X receptors-α and β [3], estrogen receptors α and β [3], [4] cytoplasmic oxysterol-binding protein (OSBP) [5], OSBP-related proteins (ORPs) [6] or insulin-induced gene (Insig) proteins [3], [7]. These interactions can not only affect sterol and lipid metabolism, they may also influence pathological processes as diverse as Niemann-Pick C disease [8] and atherosclerosis [2].

The signaling network of oxidized (phospho)lipids and their derivatives is complex. Many structurally related, biologically active compounds are generated from arachidonic acid *via* the eicosanoid synthesis pathway (also known as the arachidonic acid cascade) [9]–[11]. These compounds, collectively known as eicosanoids, include prostaglandins (PGs), thromboxanes (TXs), leukotrienes (LTs) and other oxidized derivatives/intermediates. Eicosanoids are key mediators and regulators of inflammatory responses [10], [12], [13] with implications for asthma [14]–[18], diabetes [19], cancer [20]–[23], colitis [24], [25], rheumatoid arthritis [26], [27], inflammatory bowel disease [28] and atherosclerosis [29], [30].

Evidence gathered over past two decades has shown that inflammation is a key player in pathophysiology of atherogenic cardiovascular disease (CVD) [31]–[33]. Consequently, therapies targeting inflammatory response have already been implemented in clinical practice of CVD [34]–[36] and further strategies and anti-inflammatory treatment regimens are being investigated [32]. An emerging approach in treating cardiovascular disease is based on using apoA-I mimetic peptides [37]–[41] with anti-inflammatory properties to sequester oxysterols and oxidized lipids [42]. These peptides have properties similar to apoA-I Milano [17], [43], a naturally occurring apoA-I mutant containing an extra cysteine disulfide bridge. ApoA-I Milano reduced atheromas by up to 30% [44] and ameliorated plaque build-up in arterial walls [45]. Whereas apoA-I Milano requires intravenous administration, D-4F (Ac-DWFKAFYDKVAEKFKEAF-NH_2_), a prominent apoA-I-mimetic peptide, can be taken orally [37], [39], [46]–[48].

This report describes OxP-11D, a novel 14-residue peptide whose sequence closely resembles a cholesterol-binding domain found in a family of pore-forming bacterial exotoxins. The ability of OxP-11D to bind oxidized phospholipids and sterols resemble those of D-4F [42]. In addition, OxP-11D reduced the release of monocyte chemotactic factors from LDL-stimulated human aortic endothelial cells, another property of D-4F. Although more extensive development of OxP-11D and related oxpholipins remains to be done, the current findings seem interesting enough to warrant its description now.

## Results

### Design and synthesis of “Oxpholipin” (OxP) peptides

**Table 1** shows the highly conserved, cholesterol-binding domain found in seven different cholesterol-dependent cytolysins, and **Table 2** shows the analogs included in this study. We noticed that modifying cysteine-3 with a bulky hydrophobic group (OxP-2) enhanced the peptide's ability to inhibit CDC-mediated hemolysis (data not shown). Accordingly, OxPs-5 to 12 were designed to introduce other substitutions at position-3, and OxPs-13 to 20 were designed to vary the separation of hydrophobic and ionic residues. We also synthesized three disulfide-linked dimers (OxP-3, OxP-3D and OxP-21) and created one trimer (OxP-22) by S-alkylation with TMEA. We completed this panel with three analogues (OxP-23 to 25) that contained α,α-di-substituted amino acids, intended to enhance resistance to proteolysis [49].

**Table 1:** Comparison of the cholesterol binding domains of selected CDC toxins.

**ALO**^443^GSGKDKTAHYSTVIPLPPNSKNIKIVAR**ECTGLAWEWWR**TIINEQNVPLTNE^494^
**PFO**^401^GNYQDKTAHYSTVIPLEANARNIRIKAR**ECTGLAWEWWR**DVISEYDVPLTNN^452^
**LLO**^455^ENNKSKLAHFTSSIYLPGNARNINVYAK**ECTGLAWEWWR**TVIDDRNLPLVKN^506^
**ALV**^432^GNWRDRSAHFSTEIPLPPNAKNIRIFAR**ECTGLAWEWWR**TVVDEYNVPLASD^483^
**SLO**^481^NNWYSKTSPFSTVIPLGANSRNIRIMAR**ECTGLAWEWWR**KVIDERDVKLSKE^532^
**IVL**^454^ENDKDKLAHFTTSIYLPGNARNINIHAK**ECTGLAWEWWR**TVVDDRNLPLVKN^505^
**PLY**^399^RNGQDLTAHFTTSIPLKGNVRNLSVKIR**ECTGLAWEWWR**TVYEKTDLPLVRK^450^
**OxP-1** R**ECTGLAWEWWR**TV

Abbreviations: ALO, anthrolysin O, from *Bacillus anthracis*; PFO, perfringolysin O, from *Clostridium perfringens*; LLO, listeriolysin O, from *Listeria monocytogenes*; ALV, alveolysin, from *Bacillus alvi*; SLO, streptolysin O, from Group A streptococcus; IVL, ivanolysin from *Listeria ivanovi*; PLY, pneumolysin from *S. pneumoniae*; OxP-1, Oxpholipin-1 (this manuscript).

**Table 2:** Sequences of Oxpholipins.

Peptide	Sequence
OxP-1	RE-Cys-Thr-G-Leu-Ala-Trp-E-Trp-Trp-RT-Val-NH_2_
OxP-2	RE-Ctb-Thr-G-Leu-Ala-Trp-E-Trp-Trp-RT-Val-NH_2_
OxP-3	RE-Cys-Thr-G-Leu-Ala-Trp-E-Trp-Trp-RT-Val-NH_2_ **I** RE-Cys-Thr-G-Leu-Ala-Trp-E-Trp-Trp-RT-Val-NH_2_
OxP-3D	RE-Cys-Thr-G-Leu-Ala-Trp-E-Trp-Trp-RT-Val-NH_2_ **I** RE-Cys-Thr-G-Leu-Ala-Trp-E-Trp-Trp-RT-Val-NH_2_
OxP-4	WA-Arg-Thr-V-Trp-Gly-Arg-L-Ctb-Glu-TE-Trp-NH_2_
OxP-4D	WA-Arg-Thr-V-Trp-Gly-Arg-L-Ctb-Glu-TE-Trp-NH_2_
OxP-5	RE-Ser-Thr-G-Leu-Ala-Trp-E-Trp-Trp-RT-Val-NH_2_
OxP-5D	RE-Ser-Thr-G-Leu-Ala-Trp-E-Trp-Trp-RT-Val-NH_2_
OxP-6	RE-Chg-Thr-G-Leu-Ala-Trp-E-Trp-Trp-RT-Val-NH_2_
OxP-7	RE-Cbl-Thr-G-Leu-Ala-Trp-E-Trp-Trp-RT-Val-NH_2_
OxP-8	RE-PhF-Thr-G-Leu-Ala-Trp-E-Trp-Trp-RT-Val-NH_2_
OxP-9	RE-Trp-Thr-G-Leu-Ala-Trp-E-Trp-Trp-RT-Val-NH_2_
OxP-10	RE-Bip-Thr-G-Leu-Ala-Trp-E-Trp-Trp-RT-Val-NH_2_
OxP-11	RE-Dpa-Thr-G-Leu-Ala-Trp-E-Trp-Trp-RT-Val-NH_2_
OxP-11D	RE-Dpa-Thr-G-Leu-Ala-Trp-E-Trp-Trp-RT-Val-NH_2_
OxP-12	RE-Ant-Thr-G-Leu-Ala-Trp-E-Trp-Trp-RT-Val-NH_2_
OxP-13	Aib-RE-Ctb-Val-R-Leu-Val-Trp-E-Trp-Trp-RE-Val-NH_2_
OxP-13D	Aib-RE-Ctb-Val-R-Leu-Val-Trp-E-Trp-Trp-RE-Val-NH_2_
OxP-14	Nic-RE-Ctb-Val-R-Leu-Val-Trp-E-Trp-Trp-RE-Val-NH_2_
OxP-14D	Nic-RE-Ctb-Val-R-Leu-Val-Trp-E-Trp-Trp-RE-Val-NH_2_
OxP-15	Nic-bA-RE-Ctb-Val-R-Leu-Val-Trp-E-Trp-Trp-RE-Val-NH_2_
OxP-15D	Nic-bA-RE-Ctb-Val-R-Leu-Val-Trp-E-Trp-Trp-RE-Val-NH_2_
OxP-16	Aib-RE-Ctb-Chg-R-Cha-Chg-Trp-E-Trp-Trp-RE-Chg-NH_2_
OxP-16D	Aib-RE-Ctb-Chg-R-Cha-Chg-Trp-E-Trp-Trp-RE-Chg-NH_2_
OxP-17	Ach-RE-Ctb-Chg-R-Cha-Chg-Trp-E-Trp-Trp-RE-Chg-NH_2_
OxP-18	Aib-RE-Ctb-Chg-R-Cha-Chg-Trp-E-Trp-Trp-RE-Chg-K-NH_2_
OxP-18D	Aib-RE-Ctb-Chg-R-Cha-Chg-Trp-E-Trp-Trp-RE-Chg-K-NH_2_
OxP-19	Aib-RE-Ctb-Ctb-R-Ctb-Ctb-Trp-E-Trp-Trp-RE-Ctb-NH_2_
OxP-20	Aib-RE-Ctb-Chg-R-Cha-Chg-Nal-E-Nal-Nal-RE-Chg-X
OxP-21	Aib-RE-Ctb-Chg-R-Cha-Chg-Nal-E-Nal-Nal-RE-Chg-X_2_
OxP-22	Aib-RE-Ctb-Chg-R-Cha-Chg-Nal-E-Nal-Nal-RE-Chg-X_3_
OxP-23	Ach-RE-Ctb-Ach-R-Leu-Ach-Trp-E-Trp-Trp-RE-Ach-NH_2_
OxP-24	Aib-RE-Ctb-Aib-R-Leu-Aib-Trp-E-Trp-Trp-RE-Aib-NH_2_
OxP-25	Aib-RE-Ctb-Ach-R-Leu-Ach-Trp-E-Trp-Trp-RE-Aib-NH_2_

Analogues whose identifiers end with a D are composed of D-amino acids. Abbreviations: Nic- nicotinic acid, PhF-1,2,3,4,5-pentafluoro-phenyl-alanine, Aib-aminoisobutyric acid, Bip-biphenyl-alanine, bA-β-alanine, Dpa-3,3′-diphenyl-alanine, Ach-1-amino-1-cyclohexane carboxylic acid, Ant-3-(9-anthryl)-alanine, Ctb-S-t-butyl-cysteine, Cha-cyclohexyl-alanine, Cbl-S-(4-methyl)benzyl-cysteine, Nal-3-(1-naphthyl)-alanine, Chg-cyclohexyl-glycine, X-(Lys-Arg)_3_-Lys-NHCH_2_CH_2_SH, X_2_-dimerized via disulphide bond, X_3_-trimerized with TMEA- tris-[2-maleimidoethyl]amine hydrochloride. Some natural amino acids are shown in standard, single letter code (e.g., R and E = arginine and glutamic acid).

### Anti-inflammatory activity of OxP peptides

Because monocyte chemotactic assays had predictive value in assessing prospective apoA-I mimetic peptides [38], we tested the ability of the Oxpholipins to suppress LDL-induced monocyte chemotaxis in cultured human aortic endothelial cells (HAEC) [50]. LDL containing oxidized phospholipids derived from arachidonic acid induces the production of monocyte chemoattractant protein-1 (MCP-1) by HAEC, an effect that may contribute to atherogenesis [50]. ApoA-I mimetic peptides such as D-4F and ApoJ inhibit LDL-induced chemotaxis *in vitro* and *in vivo*, by sequestering or otherwise removing lipid hydroperoxides [51], [52].

**Figure 1:**
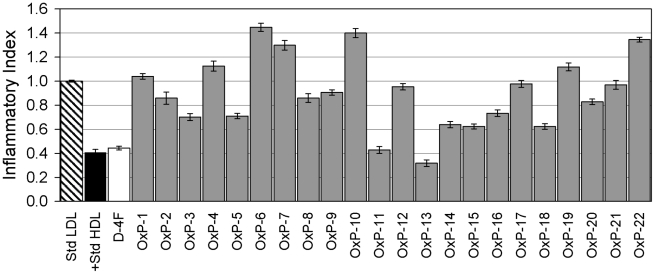
Monocyte chemotaxis assay of OxP peptides. Results were standardized to LDL and are expressed as Inflammatory Index (II). Agents with an II ≈1 are considered to be inactive. Agents with an II>1 are considered to be pro-inflammatory and those with an II<1 are considered to be anti-inflammatory. OxP-11, OxP-13 and D-4F showed very similar activity.

**Figure 2:**
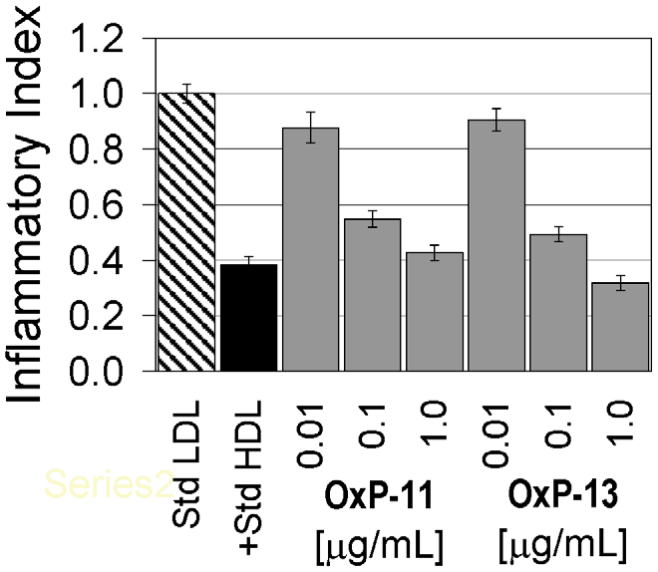
Dose response experiments. OxP-11 and OxP-13, the two most active oxpholipins in the monocyte chemotaxis assay were re-tested at three concentrations: 1 µg/ml, 0.1 µg/ml and 0.01 µg/ml. A 10-fold reduction, from 1.0 to 0.1 µg/ml caused a relatively small decrease in activity, but a 100-fold reduction to 10 ng/ml reduced activity substantially.

When tested at 1 µg/ml, several Oxpholipins reduced the monocyte-chemotactic activity of LDL (**Figure 1**). The most active peptides, OxP-11 and 13, had activity comparable to D-4F. Tests performed at lower concentrations: (0.01, 0.1 and 1 µg/ml) showed their effects to be dose-dependent (**Figure 2**). Based on these initial monocyte chemotaxis assays, we synthesized eight additional peptides (OxPs-3D, 5D, 11D, 13D, 14D, 15D, 16D and 18D), all composed exclusively of D-amino acids, hoping to obtain analogs that would be effective after oral administration (not yet tested).

**Figure 3:**
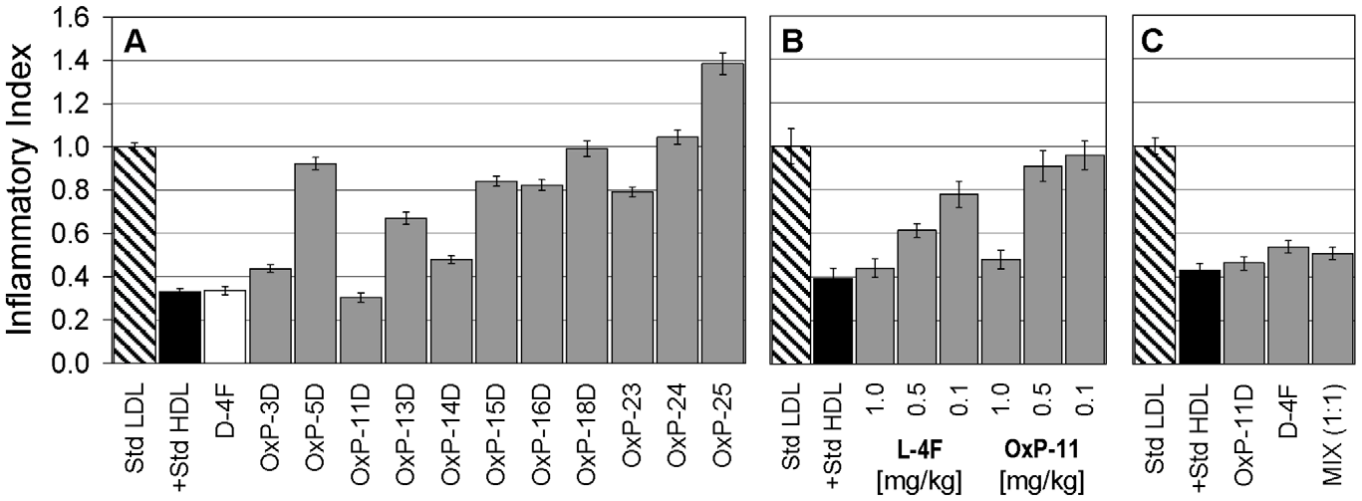
*In vivo* activity of selected OxP peptides. Panel A: Fasting female apoE deficient mice received 1.0 mg/kg of OxP peptides subcutaneously in 200 µL of ABCT buffer. After 6 hours of additional fasting, blood was obtained and HDL was isolated and tested in the monocyte chemotaxis assay as described in Methods. Results were normalized to LDL and are expressed as Inflammatory Indices. An index below 1.0 indicates anti-inflammatory activity in this test. Panel B compares L-4F and OxP-11 both containing L-amino acids exclusively. Both peptides were injected subcutaneously at three different amounts, 1.0, 0.5 and 0.1 mg/kg. Panel C compares the *in vivo* activity of D-4F, OxP-11D and a 1∶1 mixture (by weight), all administered at 1 mg/kg. D-4F and OxP-11D were equally effective alone and in combination.

When the analogues were administered subcutaneously to apoE deficient mice at 1 mg/kg (**Figure 3A**), several showed anti-inflammatory activity, including OxP-11D whose activity rivaled D-4F. Given subcutaneously, L-4F (the all L-amino acid version of D-4F) is also active in this apoE-deficient mouse model. Accordingly, we gave 3 different concentrations (0.1, 0.5 and 1 mg/kg) of all-L OxP-11 subcutaneously to apoE deficient mice (**Figure 3B**). L-4F and OxP-11 had similar efficacy at 1 mg/kg, but OxP-11 was considerably less active than L-4F at the lower concentrations.

We speculated that if the anti-inflammatory effects of D-4F and OxP-11D on monocyte chemotaxis arose from different mechanisms, synergism might ensue when the peptides were given in combination. However, we saw no apparent *in vivo* synergy between D-4F and OxP-11D at 1 mg/kg dose (**Figure 3C**). Lower doses were not tested.

### OxP-11D binds oxidized lipids and sterols

**Figure 4:**
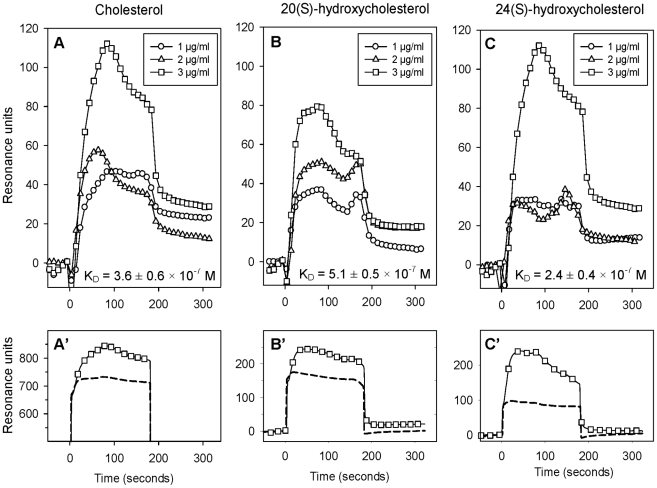
Binding of OxP-11D to cholesterol, 20(S)- and 24(S)-hydroxycholesterols. In panels A', B' and C' the dashed line shows binding of 3 µg/ml of the lipid to the biosensor's matrix and the line decorated with square symbols shows its binding to the sensor presenting OxP11-D. In panels A, B, and C, the results show binding isotherms corrected for background binding to the matrix.

**Figure 5:**
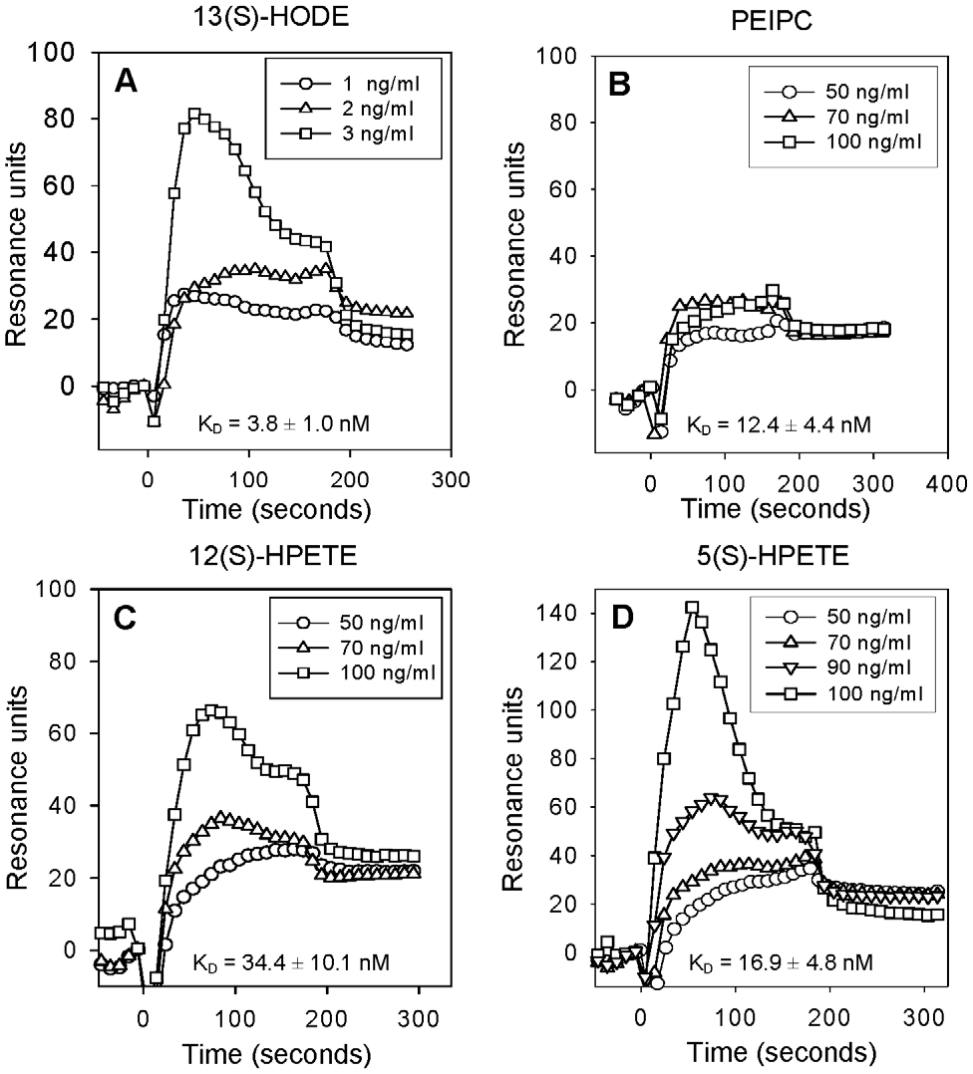
Binding of oxidized lipids to OxP-11D. Abbreviations: 13(S)-HODE: 13(S)-hydroxyoctadecadienoic acid, PEIPC: 1-palmitoyl-2-(5,6-deoxyisoprostane E2)-sn-glycero-3-phosphorylcholine, 12(S)-HPETE: 12(S)-hydroperoxyeicosatetraenoic acid, 5(S)-HPETE: 5(S)-hydroperoxyeicosatetraenoic acid. The biosensor presented 4686 RU of OxP-11D.

We used surface plasmon resonance (SPR) to examine binding of OxP-11D to various sterols, oxidized and non-oxidized lipids. Binding of some of our peptides to cholesterol, including OxP-11D, was suggested by our early experiments showing that peptides can prevent hemolysis of human RBCs mediated by CDCs in concentration dependent manner (data not shown, separate manuscript in preparation) and we decided to investigate this phenomenon in greater detail. OxP-11D binds to cholesterol (**Figure 4A**) and several oxysterols, including 20(*S*)-hydroxycholesterol (**Figure 4B**) and 24(*S*)-hydroxycholesterol (**Figure 4C**). D-4F and OxP-11D bound these sterols similarly (**Table 3**). **Figure 5 A-D** shows binding isotherms for OxP-11D to 13(*S*)-HODE), PEIPC, 12(*S*)-HPETE, and 5(*S*)-HPETE. OxP-11D bound five of the lipids with higher affinity than D-4F, including palmitic acid, 5(*S*)-HPETE, 12(*S*)-HPETE, 15(*S*)-HPETE, 13(*S*) and 13(*S*)-HODE. Ten lipids were bound with higher affinity by D-4F, including arachidonic and linoleic acids, PGPC, POVPC, PEIPC, Kodia-PC, 13(*S*)-HPODE, 12(*S*)-HETE, 15(*S*)-HETE and 9(*S*)-HODE (**Table 3**).

**Table 3:** Binding of OxP-11D and D-4F to oxidized and nonoxidized (phospho)lipids and sterols.

Ligands		K_D_ ± SEM (M)		D-4F/OxP-11D
	apoA-I	D-4F	OxP-11D	K_D_ Ratio
*Nonoxidized lipids*				
Arachidonic acid	1.0±0.1×10^−8^	1.2±0.1×10^−8^	9.7±3.0×10^−7^	0.012
Linoleic acid	6.5±2.5×10^−9^	1.5±0.1×10^−8^	6.0±1.3×10^−7^	0.025
Palmitic acid	2.0±0.3×10^−6^	6.7±0.7×10^−7^	3.3±0.7×10^−7^	2.030
*Oxidized lipids*				
PGPC	4.2±4.2×10^−6^	6.3±4.2×10^−9^	3.2±0.6×10^−6^	0.002
POVPC	3.1±2.8×10^−5^	2.4±2.0×10^−9^	3.0±0.4×10^−7^	0.008
PEIPC	5.1±0.6×10^−5^	6.0±5.0×10^−11^	1.2±0.4×10^−8^	0.005
KOdiA-PC	2.1±1.1×10^−5^	5.8±0.0×10^−10^	1.9±0.5×10^−7^	0.003
5(*S*)-HPETE	1.6±0.3×10^−3^	1.8±0.7×10^−8^	1.7±0.5×10^−8^	1.059
12(*S*)-HPETE	8.2±2.7×10^−4^	1.7±1.2×10^−7^	3.4±1.0×10^−8^	5.000
15(*S*)-HPETE	1.0±0.2×10^−3^	5.1±1.3×10^−8^	4.5±1.8×10^−8^	1.133
13(*S*)-HPODE	1.2±0.6×10^−3^	2.1±0.5×10^−8^	3.1±0.5×10^−8^	0.677
12(*S*)-HETE	8.5±3.3×10^−4^	1.8±0.5×10^−8^	4.8±1.2×10^−8^	0.375
15(*S*)-HETE	1.3±0.1×10^−3^	2.2±0.8×10^−8^	9.3±1.5×10^−8^	0.236
9(*S*)-HODE	1.3±0.5×10^−3^	1.5±0.4×10^−8^	2.1±0.5×10^−8^	0.714
13(*S*)-HODE	1.8±0.3×10^−3^	1.1±0.6×10^−8^	3.8±1.0×10^−9^	2.895
*Sterols*				
Cholesterol	BND	9.2±0.3×10^−8^	3.6±0.6×10^−7^	0.255
20(*S*)-Hydroxycholesterol	BND	3.9±2.1×10^−6^	5.1±0.5×10^−7^	7.647
22(*S*)-Hydroxycholesterol	BND	1.5±0.4×10^−5^	4.5±0.8×10^−7^	33.333
24(*S*)-Hydroxycholesterol	BND	4.3±1.6×10^−6^	2.4±0.4×10^−7^	17.917
25-Hydroxycholesterol	BND	2.4±1.5×10^−6^	BND	―
4β-Hydroxycholesterol	BND	3.1±1.7×10^−5^	BND	―
*Control*				
Bovine albumin	2.1±0.4×10^−8^	3.9±1.5×10^−6^	6.9±1.0×10^−7^	2.609

The mean K_D_ values for apoA-I and D-4F are from [42], and those for OxP-11D are from this study. The ratio (K_D_^D-4F^)/(K_D_^OxP-11D^) is also shown. Ratios below 1 indicate that D-4F binds the lipid with higher affinity (lower K_D_) than OxP-11D. Ratios greater than 1 indicate that OxP-11D binds the lipid with higher affinity than D-4F. Abbreviations: PGPC-1-palmitoyl-2-glutaroyl-sn-glycero-3-phosphorylcholine; POVPC-1-palmitoyl-2-(5-oxovaleroyl)-sn-glycero-3-phosphorylcholine; PEIPC-1-palmitoyl-2-(5,6-epoxyisoprostane E2)-sn-glycero-3-phosphorylcholine; KOdiA-PC-1-palmitoyl-2-(5-keto-6-octene-dioyl)-sn-glycero-phosphatidylcholine; HPETE-hydroperoxyeicosatetraenoic acid; HPODE-hydroperoxyoctadecadienoic acid, HETE- hydroxyeicosatetraenoic acid; HODE- hydroxyoctadecadienoic acid.

Generally, D-4F seems to bind with higher affinity to oxidized lipids containing palmitoyl moiety: PGPC, POVPC, PEIPC and KOdiA-PC. OxP-11D on the other hand seems to be more selective toward certain sterols: 20(*S*)-hydroxycholesterol, 22(*S*)-hydroxycholesterol, 24(*S*)-hydroxycholesterol and certain arachidonic acid derivatives: 5(*S*)-HPETE, 12(*S*)-HPETE, 15(*S*)-HPETE and 13(*S*)-HODE. The position of oxidation appeared to influence binding affinity, as OxP-11D bound tightly to cholesterol derivatives hydroxylated in positions 20(*S*)-, 22(*S*)- and 24(*S*)-, but not to 25-hydroxycholesterol or 4β-hydroxycholesterol which were preferentially bound by D-4F.

### CD and FTIR studies of secondary structure

**Figure 6:**
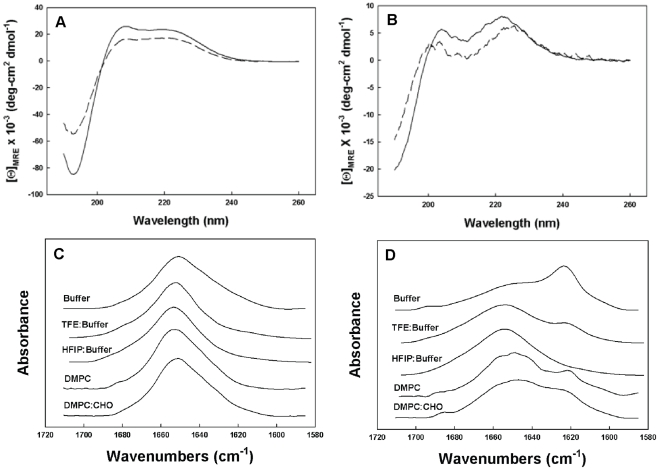
Circular dichroism (CD) and Fourier-Transform Infrared (FTIR) spectra. Panel A shows CD spectra of D-4F and Panel B shows the spectra of OxP-11D. Spectra were obtained in two solvent systems: 10 mM phosphate buffer, pH 7.4 (dashed line ----), HFIP:Buffer, (10 mM, pH 7.4) 4∶6, v:v (solid line —). The peptide concentration was 100 µM, the cuvette light path was 0.01 cm, the temperature was 25°C, and the spectra are the average of 8 scans. Panel C shows FTIR spectra of D-4F and Panel D shows FTIR spectra of OxP-11D. The spectra were obtained in D_2_O Buffer, TFE: deuterium Buffer 4∶6, v:v, HFIP: deuterium Buffer, 4∶6, v:v, deuterium vapor hydrated DMPC multilayers (peptide:lipid, 1∶10, mole:mole), and deuterium vapor hydrated DMPC:CHO (DMPC:CHO,1.2∶1, mole:mole with a peptide to lipid ratio of 1∶10, mole:mole).

D-4F and OxP-11D assumed stable conformations (**Figure 6A, 6B**) in aqueous buffer and in the structure-promoting HFIP:buffer solvent system. CD spectra of D-4F showed a highly helical structure (**Table 4**) with the definitive maxima at 222 and 208 nm expected for a mostly α-helical, D-amino acid peptide. FTIR measurements of D-4F, both in buffer and in structure-promoting solvent systems, revealed strong absorbance around 1656 cm-1 (**Figure 6C**), indicating a predominantly helical secondary structure (**Table 5**) consistent with the CD studies. In contrast, the FTIR spectra of OxP-11D in aqueous buffer showed a mixture of helical (1662-1645 cm^−1^), turn (1682-1662 cm^−1^), disordered (1650-1637 cm^−1^) and β-sheet (1637-1613 and 1710-1682 cm^−1^) structural elements. In “structure-promoting” solvents, OxP-11D showed more turn and helix propensity, suggesting it might assume a more highly ordered structure in these more amphipathic and hydrophobic environments (**Figure 6D**).

**Table 4:** Proportions of different components of secondary structure for D-4F and OxP-11D peptides based on Circular Dichroic spectroscopic analysis.

Sample*	Conformation (%)			
	α-helix	turns	β-sheet	disordered
D-4F in Buffer	65.0	12.0	13.0	10.0
D-4F in HFIP:Buffer	74.0	9.0	11.0	6.0
OxP-11D in Buffer	15.0	19.0	30.0	36.0
OxP-11D in HFIP:Buffer	25.0	33.0	14.0	28.0

* peptides (100 µM) in 10 mM phosphate buffer pH 7.4 or HFIP:10 mM phosphate buffer pH 7.4 were analyzed for secondary conformation based as described in the Methods section.

**Table 5:** Secondary structural composition of D-4F and OxP-11D in different solvent systems, inferred from infrared (IR) spectroscopy.

Peptide/Solvent System	Conformation (%)			
	α-helix	turns	β-sheet	disordered
D-4F/Buffer	63.0	11.4	11.8	13.7
D-4F/TFE:Buffer	67.2	8.8	11.3	12.7
D-4F/HFIP:Buffer	71.5	8.0	11.9	8.6
D-4F/DMPC (1∶10 mole:mole)	57.8	16.4	1.9	23.9
D-4F/DMPC:CHO (1∶10 mole:mole)	58.1	15.2	4.1	22.6
OxP-11D/Buffer	20.1	24.8	33.7	21.4
OxP-11D/TFE:Buffer	32.9	31.0	22.9	13.2
OxP-11D/HFIP:Buffer	32.4	37.3	10.3	20.0
OxP-11D/DMPC (1∶10 mole:mole)	26.5	24.4	24.9	24.2
OxP-11D/DMPC:CHO (1∶10 mole: mole)	29.5	27.7	22.6	20.2

The peptides (1 mM) were studied in the following solvent systems: deuterated 10 mM phosphate buffer pD 7.4 (“DPB”); trifluoroethanol (TFE):DPB; or hexafluoroisopropanol (HFIP): DPB. Measurements were done and spectra were analyzed as described in the Methods. The peak area error for these estimates is ±4%.

To characterize the secondary structures of both peptides in lipid environments, we performed FTIR in hydrated lipid multilayers of DMPC ±cholesterol. In multilayers composed of DMPC ± cholesterol, D-4F assumed a strong helical conformation, manifested by a dominant absorption peek at 1656 cm^−1^ similar to its spectrum in the aqueous solvent systems. OxP-11D displayed a more complex conformational signature in the multilayers. In phospholipids alone (DMPC) there were contributions from helix (1662-1645 cm^−1^) followed by turn (1682-1662 cm^−1^), disordered (1650-1637 cm^−1^) and β-sheet (1637-1613 and 1710-1682 cm^−1^) conformations respectively (**Table 4**). In multilayers of DMPC with cholesterol, OxP-11D displayed similar FTIR characteristics, but with a slightly higher helical signal and lower contributions from turn and β-sheet elements (**Table 5**).

### Secondary structure studies: molecular dynamics simulations

**Figure 7:**
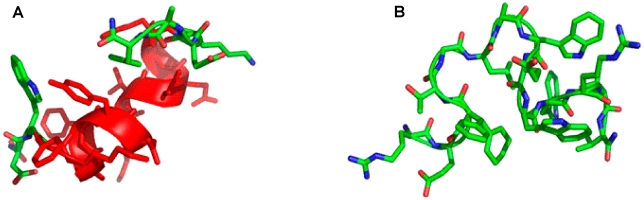
Molecular illustration of the structure of D-4F (A) and OxP-11D (B) after 83 nsec of molecular dynamics in HFIP:aqueous buffer 6∶4, v:v environment. α-helical segments are in red ribbon, disordered and turn segments are in green highlight The N-terminus is at the lower left and the adjacent C-terminus is in the upper right of the figure.

**Figure 8:**
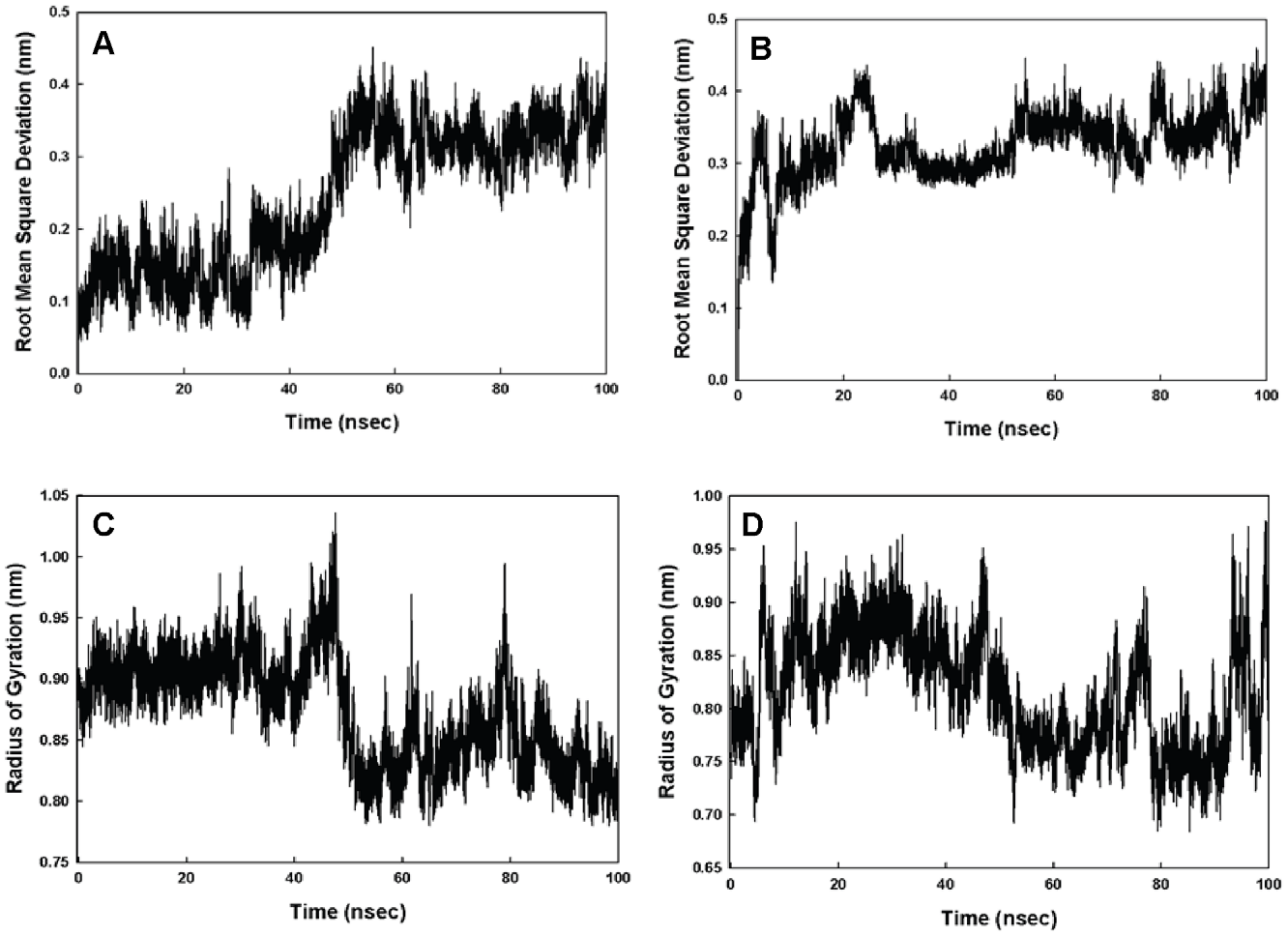
Time dependent stability studies. Panel A shows Root Mean Square Deviation (RMSD) of the C-α carbons for D-4F. Panel B shows these RMSD values for OxP-11D. Panel C shows the radius of gyration for D-4F and Panel D shows the radius of gyration for OxP-11D. All values are shown as a function of time (in nanoseconds) and come from molecular dynamic studies in a simulated HFIP:buffer (6∶4) environment.

**Figure 9:**
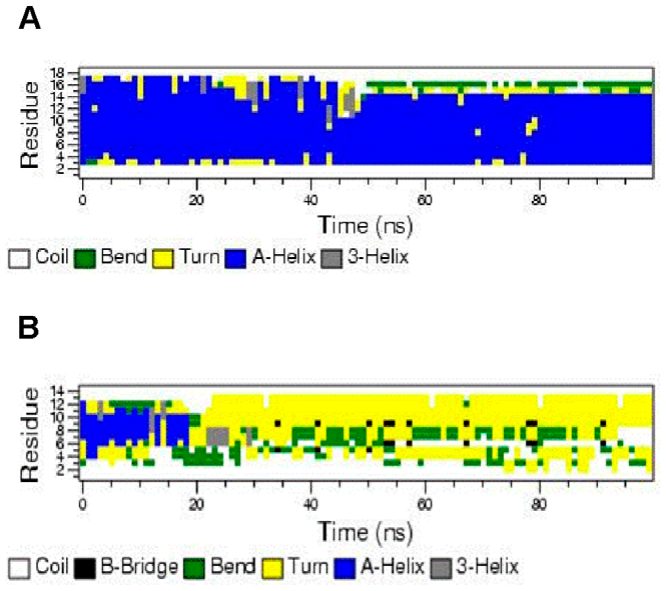
DSSP plot of secondary structure of D-4F (A) and OxP-11D (B) as a function of time in a simulated HFIP:buffer 6∶4 environment.

Simulations of D-4F and OxP-11D in the “structure-promoting” HFIP:buffer, solvent system were consistent with the structures deduced from CD and FTIR measurements (**Figure 7**). D-4F maintained a highly helical structure in the simulated HFIP:buffer environment. After 40 nanoseconds, the radius of gyration and RMSD of its C-α carbon backbone atoms indicated a stable conformation with an uninterrupted helix between residues 2 to 15. In contrast, within 20 nanoseconds, OxP-11D changed its initially helical structure to a more flexible conformation with multiple turn-like features, evident from both the C-α carbons and the radius of gyration (**Figure 8**). The DSSP plot of OxP-11D after 20 nanoseconds revealed a stable turn (residues 9 to 13), some helical propensity (residues 4–6), and more disordered-coil conformations elsewhere (**Figure 9**).

## Discussion

This study was undertaken to investigate the properties of peptides derived from a highly conserved, cholesterol-binding domain found in over 20 bacterial exotoxins (“cholesterol dependent cytolysins”) secreted by Gram-positive bacteria. Initially, we hoped only to design peptides or peptidomimetic analogs that, by binding cholesterol, would protect human cells from the lytic effects of these bacterial exotoxins, Indeed, several of the peptides in **Table 2** protected human erythrocytes from lysis by anthrolysin O, listeriolysin O, and pneumolysin. These studies will be described elsewhere.

Because peptides that bind cholesterol lipids have potential as anti-inflammatory and/or anti-atherosclerosis agents we also examined the binding of other lipids by the “Oxpholipin” peptides, as well as their activity in a monocyte chemotaxis assay that guided the initial development of L-4F and related apoA-I-mimetic peptides. The results of these studies caused us to focus on OxP-11D, which is the principal subject of this report.

*In vitro* and in studies with apoE deficient mice, the effects of OxP-11D on monocyte chemotaxis were dose-dependent and similar in magnitude to those of D-4F. Surface plasmon resonance studies revealed additional similarities to D-4F. Both peptides bound normal and oxidized cholesterol, but OxP-11D did so with higher affinity. Five of the 15 nonsteroid lipids in **Table 3** were apparently bound with higher affinity by OxP-11D and ten were bound more effectively by D-4F. For example, the affinity of D-4F for arachidonic and linoleic acid, POVPC, PEIPC and KODia-PC appear to be considerably higher than that of OxP-11D.

Certain studies suggest that the factors responsible for the different binding preferences of D-4F and OxP-11D reside in the fine structure of the lipids as well as in those of the peptides [52]–[57]. For example, OxP-11D bound 12(S)-HPETE (hydroperoxyeicosatetraenoic acid) with higher affinity than D-4F, and yet D-4F bound 12(S)-HETE (hydroxyeicosatetraenoic acid) with much higher affinity than OxP-11D. Similarly, OxP-11D bound 13(*S*)-HODE (hydroxyoctadecadienoic acid) with higher affinity than D-4F, yet D-4F had greater affinity for 9(S)-HODE.

Changes in the secondary structure of OxP-11D in lipid environments were noted in our structural studies, and may correlate with our functional measurements *in vitro* and in vivo. CD, FTIR and molecular dynamics studies showed the peptide to have considerable conformational freedom. OxP-11D had a less defined conformation in more polar environments, however in hydrophobic solvent systems and in lipid multilayer ensembles, including these with cholesterol, the peptide showed highly ordered helix and turn structures.

Recently, L-4F and D-4F were shown to bind oxidized lipids with much higher affinity than human apoA-I [42] and it was suggested that this property might contribute to its anti-inflammatory activity by sequestering strongly pro-inflammatory oxidized sterols and lipids [2], [3], [10], [13], [58], [59]. How do the lipid binding properties of D-4F and OxP-11D compare? If we can use the K_D_ values and ratios shown in **Table 3** as a basis for comparison, there are similarities and differences. Both peptides bind cholesterol and its oxidized derivatives, with the advantage going to OxP-11D. This is hardly surprising, given that 13 of the 14 residues (92.7%) in OxP-11D are identical to the library's cholesterol-binding design template.

Their respective binding of 5(S)-HPETE and 12(S)-HPETE is also instructive. 5(S)-HPETE, which is derived from arachidonic acid by the actions of 5-lipoxygenase, is a direct precursor of leukotrienes A4, B4 and C4. The K_D_ values (**Table 3**) show that D-4F binds arachidonic acid, 5(S)-HPETE and 12(S)-HPETE with similar affinities. In contrast, based on the K_D_ ratios, the affinity of OxP-11D for 5(S)-HPETE was approximately 57-fold higher than its affinity for arachidonic acid, and its affinity for 12(S)-HPETE was approximately 28.5-fold higher. Whether these differences will prove advantageous or detrimental in animal trials of OxP-11D cannot be predicted. Nonetheless, OxP-11D bound oxidized lipids and sterols preferentially, and with much higher affinity than apoA-I. Therefore it may function as oxidized-compounds' sequestering agent influencing lipid metabolism on molecular and cellular level. For these and other reasons, OxP-11D and structurally related peptides are interesting lead compounds whose further study could result in the development of novel peptide and peptidomimetic therapeutics.

## Materials and Methods

### Ethics Statement

The experiments were performed using protocols approved by the Animal Research Committee at UCLA.

### Peptide synthesis and characterization

Solid phase peptide synthesis was done with a Symphony® automated peptide synthesizer (Protein Technologies Inc., Tucson, AZ) or a CEM Liberty automatic microwave peptide synthesizer (CEM Corporation Inc., Matthews, NC), using 9-fluorenylmethyloxycarbonyl (Fmoc) chemistry [60]. Amino acid derivatives and reagents were from EMD Biosciences (San Diego, CA) or Chem-Impex International (Wood Dale, IL). After cleaving the peptides from the resin with modified reagent K (TFA 94% (v/v); phenol, 2% (w/v); water, 2% (v/v); TIS, 2% (v/v); 2 hours) they were precipitated with ice-cold diethyl ether and purified to >95% homogeneity by preparative reverse-phase high performance liquid chromatography (RP-HPLC).

Peptide purity was evaluated by matrix-assisted laser desorption ionization spectrometry (MALDI-MS) and by analytical RP-HPLC, using a ProStar 210 HPLC system with a ProStar 325 Dual Wavelength detector set at 220 nm and 280 nm (Varian Inc., Palo Alto, CA). The mobile phases were: Solvent A, 0.1% TFA in water; solvent B, 0.1% TFA in acetonitrile. Analytic assessments used a reversed-phase, 4.6(250 mm C18 column (Vydac 218TP54) and a linear 0 to 100% gradient of solvent B applied over 100 min at 1 ml/min. Peptides OxP-3, OxP-3D and OxP-21 were obtained by dimerizing the appropriate monomer (10 mg/ml) in a 50% aqueous solution of DMSO at room temperature over 48 h. OxP-22 was obtained by trimerizing OxP-20 in 50% aqueous solution of DMF using Tris-[2-maleimidoethyl]amine and the manufacturer's protocol (Pierce Biotechnology, Rockford, IL, Cat#33043). The progress of the reaction was monitored by mass spectrometry. All peptides were lyophilized for storage. Peptide stock solutions were made in HPLC grade water containing 0.01% acetic acid, and peptide concentrations were determined by absorbance at 280 nm [61]. For analytical details concerning synthesized peptides please see Supporting Information (**Table S1** and **Figure S1**).

### Lipids

PAPC (L-α-1-palmitoyl-2-arachidonoyl-*sn*-glycero-3-phosphorylcholine), PAPE (1-palmitoyl-2-arachidonoyl-*sn*-glycero-3-phosphatidylethanolamine), POVPC (1-palmitoyl-2-(5-oxovaleroyl)-*sn*-glycero-3-phosphorylcholine, PGPC (1-palmitoyl-2-glutaroyl-*sn*-glycero-3-phosphorylcholine), and POPC (1-Palmitoyl-2-oleoyl-*sn*-glycero-3-phosphorylcholine) were from Avanti Polar Lipids (Alabaster, AL). Cholesterol, ≥99% pure, 20(*S*)-hydroxycholesterol, 22(*S*)- and 25(*S*)-hydroxycholesterol, arachidonic acid, linoleic acid, palmitic acid and bovine serum albumin were from Sigma-Aldrich (St. Louis, MO). 13(*S*)-HPODE (hydroperoxyoctadecadienoic acid), 5(*S*)-, 12(*S*)-, and 15(*S*)-HPETE (hydroperoxyeicosatetraenoic acid), 12(*S*)- and 15(*S*)-HETE (hydroxyeicosatetraenoic acid), were from BioMol, Plymouth Meeting, PA. 9(*S*)- and 13(*S*)-HODE (hydroxyoctadecadienoic acid). KOdiA-PC was from Cayman Chemical US (Ann Arbor, MI). 24(*S*)-hydroxycholesterol was from Steraloids (Newport, RI). PEIPC (1-palmitoyl-2-(5,6-deoxyisoprostane E2)-*sn*-glycero-3-phosphorylcholine) was prepared as previously described [11], [62].

### Binding studies

Binding experiments were done by surface plasmon resonance (SPR) on a BIAcore 3000 system (BiaCore AB, Piscataway, NJ). Peptide ligands and apoA-I were immobilized on a BIAcore CM5 sensor chip activated per the manufacturer's protocol with N-hydroxysuccinimide and 1-ethyl-3-(3-dimethylaminoisopropyl) carbodiimide. After achieving adequate immobilization, the activated sensor surface was blocked with ethanolamine.

Lipid stock solutions were prepared in absolute ethanol and then diluted into a standard BIAcore buffer (HBS-EP), containing 10 mM HEPES, pH 7.4, 150 mM NaCl, 3 mM EDTA and 0.005% (v/v) surfactant P20. Lipid concentrations used in the binding studies were selected to give binding responses of 30-500 resonance units. Lipid stock solutions were prepared at 1 mg/ml in ethanol. Since the highest analyte lipid concentrations did not exceed 10 µg/ml, the highest ethanol concentration in any analyte solutions was 1%, and for most lipids the ethanol concentration was considerably lower. Ethanol-free HBS-EP buffer was used during the dissociation phase. Lipid binding was measured by observing the change in the SPR angle as 150 µl of lipid analyte (various concentrations) in HBS-EP buffer flowed over the biosensor for 3 min at 50 µl/min. Biosensors were washed with 25 or 50% ethanol to regenerate them between binding studies. SPR data were corrected for background binding to the matrix of the chip (“blank” channel) and analyzed with BIAevaluation 4.1 software (Biacore, Piscataway, NJ).

### Human monocytes and aortic endothelial cells

Normal human monocytes were isolated as previously described [63]. Human aortic endothelial cells (HAEC) were isolated and maintained as previously reported [64]. Acquisition and use of these cells was in accordance with protocols approved by the UCLA Human Research Subject Protection Committee.

### Monocyte chemotaxis assays

As previously described [63], [64], HAEC cells were treated with native LDL (250 µg/ml) in the absence or presence of HDL or tested peptides for 8 h. After these cultures were washed, the medium was replaced by fresh Medium 199 and the cultures were incubated for an additional 8 h. This culture medium was collected and assayed for monocyte chemotactic activity using chambers purchased from Neuroprobe (Cabin John, MD). After monocytes were added to the upper compartment, the chamber was incubated for 60 min at 37°C and subsequently disassembled. The membrane was rinsed, air dried, fixed with 1% glutaraldehyde, stained with 0.1% crystal violet dye, and 12 standardized high power fields were examined microscopically. The number of migrated monocytes was expressed as the mean ±SD of monocytes counted. Values obtained in the absence of HDL were normalized to a value of 1.0. Normalized values >1.0 after HDL addition were considered to be pro-inflammatory, and values <1.0 as being anti-inflammatory.

### Mouse experiments

ApoE null mice on a C57BL/6J background, originally from Jackson Laboratories (Bar Harbor, ME), were maintained in a breeding colony in the Department of Laboratory and Animal Medicine at the David Geffen School of Medicine at UCLA. The mice were maintained on a chow diet (Ralston Purina, St. Louis, MO).

Groups of 6 fasting female apoE deficient mice, 4–6 months of age, were injected subcutaneously with 200 µL of either ABCT buffer (50 mM NH_4_HCO_3_, 0.1% Tween 20) or 1.0 mg/kg of OxP peptides in ABCT buffer. Six hours later, and with continued fasting, blood was removed from the retro-orbital sinus under mild isoflurane anesthesia, and anticoagulated with heparin (2.5 U/ml). Plasma was obtained and fractionated by FPLC. HDL-containing fractions were tested in HAEC cultures for their ability to inhibit LDL-induced monocyte chemotactic activity. All procedures conformed to regulations of the UCLA Animal Research Committee. An HDL inflammatory index was determined using the standard monocyte chemotaxis assay. Indices >1 were interpreted as pro-inflammatory, and indices <1 as anti-inflammatory.

### Circular dichroism (CD) analyses of secondary structure

CD spectra from 190–260 nm of D-4F and OxP-11D were examined in different solution environments using a JASCO 715 spectropolarimeter (Jasco Inc., Easton, MD) that was calibrated for wavelength and optical rotation with 10-camphorsulphonic acid [65], [66]. Peptides were scanned at 20 nm per minute in 0.01 cm path-length cells at 25°C with a sample interval of 0.2 nm. Peptide concentration was determined by UV absorbance at 280 nm. After baseline correction, the spectra were expressed as the Mean Residue Ellipticity [θ]_MRE_. Quantitative estimates of the secondary structural contributions were made with VARSLC [67] using the spectral basis set for proteins implemented in the Olis Global Works™ software package (Olis Inc., Bogart, GA).

### Fourier transform infrared (FTIR) spectroscopy

Infrared spectra were recorded at 25°C using a Bruker Vector 22™ FTIR spectrometer with a deuterated triglycine sulfate (DTGS) detector, and averaged over 256 scans at a gain of 4 with a resolution of 2 cm^−1^. Lipid and peptide samples were initially freeze-dried several times from 10 mM HCl in D_2_O to remove any interfering counter ions and residual H_2_O. Solution spectra of peptides were made in deuterated 10 mM phosphate buffer, pD 7.4 (pD  =  pH+0.4) and in structure promoting mixed solvent-buffer solutions (trifluoroethanol (TFE) or hexafluoroisopropanol (HFIP) at a sample concentration of 1 mM.

Spectra were acquired using a temperature controlled, demountable liquid cell with calcium fluoride windows fitted with a 50 µm thick spacer (Harrick Scientific, Pleasantville, NY). Lipid-peptide films were prepared by air-drying mixtures of DMPC and DMPC:cholesterol (1.2∶1, mole:mole) in chloroform with D-4F or OxP-11D in TFE onto a 50×20×2 mm, 45° ATR crystal (Pike Technologies, Madison, WI) fitted to the Bruker spectrometer [68] to form a multilayer film (lipid:peptide, 10∶1, mole:mole). After evaporation the solvent lipid:peptide film was hydrated by passaging deuterium-saturated nitrogen gas through the sample chamber for one hour prior to spectroscopy [69]–[71]. The relative proportions of α-helix, β-turn, β-sheet, and disordered conformations of solution and multilayer IR spectra were determined by Fourier self-deconvolution for band narrowing and area calculations of component peaks of the FTIR spectra using curve-fitting software supplied by Galactic Software (GRAMS/AI, version 8.0; Thermo Electron Corp., Waltham, MA). The frequency limits for the different structures were: α-helix (1662-1645 cm^−1^), β-sheet (1637-1613 and 1710-1682 cm^−1^), turns (1682-1662 cm^−1^), and disordered or random (1650-1637 cm^−1^) [72].

### Molecular dynamics modeling

Monomeric starting structures for D-4F and OxP-11D were obtained by using Hyperchem 7.5 (http://www.hyper.com) to build the peptides in a helical conformation. These structures were placed in a periodic 56 Å^3^ box of TIP4P water or HFIP:TIP4P water (4∶6, v:v) and the ensemble was neutralized with counter-ions to simulate the environment used for our CD measurements (equilibrated HFIP solvent box and topology files courtesy of D. Roccatano) [73]. The peptide in the solution box was conjugate-gradient- minimized using the Polak-Ribiere approach implemented in Hyperchem. Minimized monomeric D-4F or OxP-11D ensembles were ported to the Gromacs program suite, version 4.0.4 (http://www.gromacs.org), and subjected to the steepest descent method using the OPLS_AA option [74].

The system was subjected to 20 psec of pre-run molecular dynamics at 300°K allowing the solvent to equilibrate while restraining the peptide. After pre-run solvent equilibration the peptides were subjected to 100 nsec of free MD simulations at 300°K without any experimental constraints, utilizing Berendsen temperature and pressure coupling and the Particle Mesh Ewald method for evaluating long-range electrostatic interactions. The time-dependent evolution of the root mean square deviations (RMSD) for the peptide C-α carbons, radius of gyration and secondary structure (i.e., analyzed using the DSSP criteria [75] for the peptide in the HFIP-water environment indicated when equilibrium was reached. Molecular model illustrations were rendered using PyMOL v0.99 (http://www.pymol.org).

## Supporting Information

Table S1 Analytical data for OxP peptides. Peptide purity was evaluated by matrix-assisted laser desorption ionization spectrometry (MALDI-MS) and by analytical RP-HPLC, using a ProStar 210 HPLC system with a ProStar 325 Dual Wavelength detector set at 220 nm and 280 nm (Varian Inc., Palo Alto, CA). The mobile phases were: Solvent A, 0.1% TFA in water; solvent B, 0.1% TFA in acetonitrile. Analytic assessments used a reversed-phase, 4.6×250 mm C18 column (Vydac 218TP54) and a linear 0 to 100% gradient of solvent B applied over 100 min at 1 ml/min.
(0.06 MB DOC)

Figure S1 Two examples of analytical RP-HPLC profiles and corresponding MS spectra obtained for OxP peptides. Panel A - OxP-5; Panel B - OxP-11D.
(0.13 MB DOC)
