# Supplementary material for: Oxpholipin 11D: An Anti-Inflammatory Peptide That Binds Cholesterol and Oxidized Phospholipids
Source: PLoS One. 2010 Apr 14;5(4):e10181. doi: 10.1371/journal.pone.0010181 (PMC2854715; doi:10.1371/journal.pone.0010181)
Supplement: Table S1 — Analytical data for OxP peptides. Peptide purity was evaluated by matrix-assisted laser desorption ionization spectrometry (MALDI-MS) and by analytical RP-HPLC, using a ProStar 210 HPLC system with a ProStar 325 Dual Wavelength detector set at 220 nm and 280 nm (Varian Inc., Palo Alto, CA). The mobile phases were: Solvent A, 0.1% TFA in water; solvent B, 0.1% TFA in acetonitrile. Analytic assessments used a reversed-phase, 4.6×250 mm C18 column (Vydac 218TP54) and a linear 0 to 100% gradient of solvent B applied over 100 min at 1 ml/min. (0.06 MB DOC) [file pone.0010181.s001.doc]

| **Peptide** | **Composition** | **MW (g/mole) Calc / Found** | **R_T_ (min)** |
| --- | --- | --- | --- |
| OxP-1 | C_82_H_118_N_24_O_20_S_1_ | 1792.07 / 1792.53 | 37.703 |
| OxP-2 | C_86_H_126_N_24_O_20_S_1_ | 1848.18 / 1848.08 | 39.619 |
| OxP-3 | C_164_H_234_N_48_O_40_S_2_ | 3582.12 / 3583.93 | 41.561 |
| OxP-3D | C_164_H_234_N_48_O_40_S_2_ | 3582.12 / 3582.93 | 41.200 |
| OxP-4 | C_86_H_126_N_24_O_20_S_1_ | 1848.18 / 1849.27 | 39.494 |
| OxP-4D | C_86_H_126_N_24_O_20_S_1_ | 1848.18 / 1848.80 | 39.305 |
| OxP-5 | C_82_H_118_N_24_O_21_ | 1776.00 / 1776.17 | 36.927 |
| OxP-5D | C_82_H_118_N_24_O_21_ | 1776.00 / 1776.01 | 37.030 |
| OxP-6 | C_87_H_126_N_24_O_20_ | 1828.12 / 1828.99 | 39.550 |
| OxP-7 | C_90_H_126_N_24_O_20_S_1_ | 1896.22 / 1897.16 | 41.630 |
| OxP-8 | C_88_H_117_N_24_O_20_F_5_ | 1926.05 / 1926.44 | 41.061 |
| OxP-9 | C_90_H_123_N_25_O_20_ | 1875.14 / 1876.37 | 39.847 |
| OxP-10 | C_94_H_126_N_24_O_20_ | 1912.20 / 1912.56 | 42.860 |
| OxP-11 | C_94_H_126_N_24_O_20_ | 1912.20 / 1912.78 | 41.157 |
| OxP-11D | C_94_H_126_N_24_O_20_ | 1912.20 / 1913.05 | 41.428 |
| OxP-12 | C_96_H_126_N_24_O_20_ | 1936.22 / 1936.14 | 42.980 |
| OxP-13 | C_98_H_148_N_28_O_21_S_1_ | 2086.51 / 2087.29 | 44.044 |
| OxP-13D | C_98_H_148_N_28_O_21_S_1_ | 2086.51 / 2086.80 | 43.624 |
| OxP-14 | C_100_H_144_N_28_O_21_ S_1_ | 2106.50 / 2107.52 | 48.715 |
| OxP-14D | C_100_H_144_N_28_O_21_ S_1_ | 2106.50 / 2106.81 | 48.445 |
| OxP-15 | C_103_H_149_N_29_O_22_ S_1_ | 2177.58 / 2178.86 | 47.310 |
| OxP-15D | C_103_H_149_N_29_O_22_ S_1_ | 2177.58 / 2178.19 | 47.311 |
| OxP-16 | C_110_H_164_N_28_O_21_ S_1_ | 2246.77 / 2247.06 | 52.401 |
| OxP-16D | C_110_H_164_N_28_O_21_S_1_ | 2246.77 / 2246.88 | 51.936 |
| OxP-17 | C_113_H_168_N_28_O_21_S_1_ | 2286.84 / 2287.19 | 55.466 |
| OxP-18 | C_116_H_176_N_30_O_22_S_1_ | 2374.95 / 2375.54 | 46.691 |
| OxP-18D | C_116_H_176_N_30_O_22_S_1_ | 2374.95 / 2375.49 | 48.480 |
| OxP-19 | C_105_H_162_N_28_O_21_S_5_ | 2312.96 / 2312.74 | 48.030 |
| OxP-20 | C_160_H_255_N_45_O_28_S_2_ | 3321.23 / 3323.91 | 52.558 |
| OxP-21 | C_320_H_508_N_90_O_56_S_4_ | 6640.44 / 6642.01 | 57.240 |
| OxP-22 | C_498_H_783_N_139_O_90_S_6_ | 10350.05 / 10351.83 | 56.788 |
| OxP-23 | C_107_H_158_N_28_O_21_S_1_ | 2204.69 / 2204.78 | 50.457 |
| OxP-24 | C_95_H_142_N_28_O_21_S_1_ | 2044.43 / 2045.18 | 40.947 |
| OxP-25 | C_101_H_150_N_28_O_21_S_1_ | 2124.56 / 2124.28 | 45.826 |
