# Supplementary figures and images for: Oxpholipin 11D: An Anti-Inflammatory Peptide That Binds Cholesterol and Oxidized Phospholipids
Source: PLoS One. 2010 Apr 14;5(4):e10181. doi: 10.1371/journal.pone.0010181 (PMC2854715; doi:10.1371/journal.pone.0010181)

| 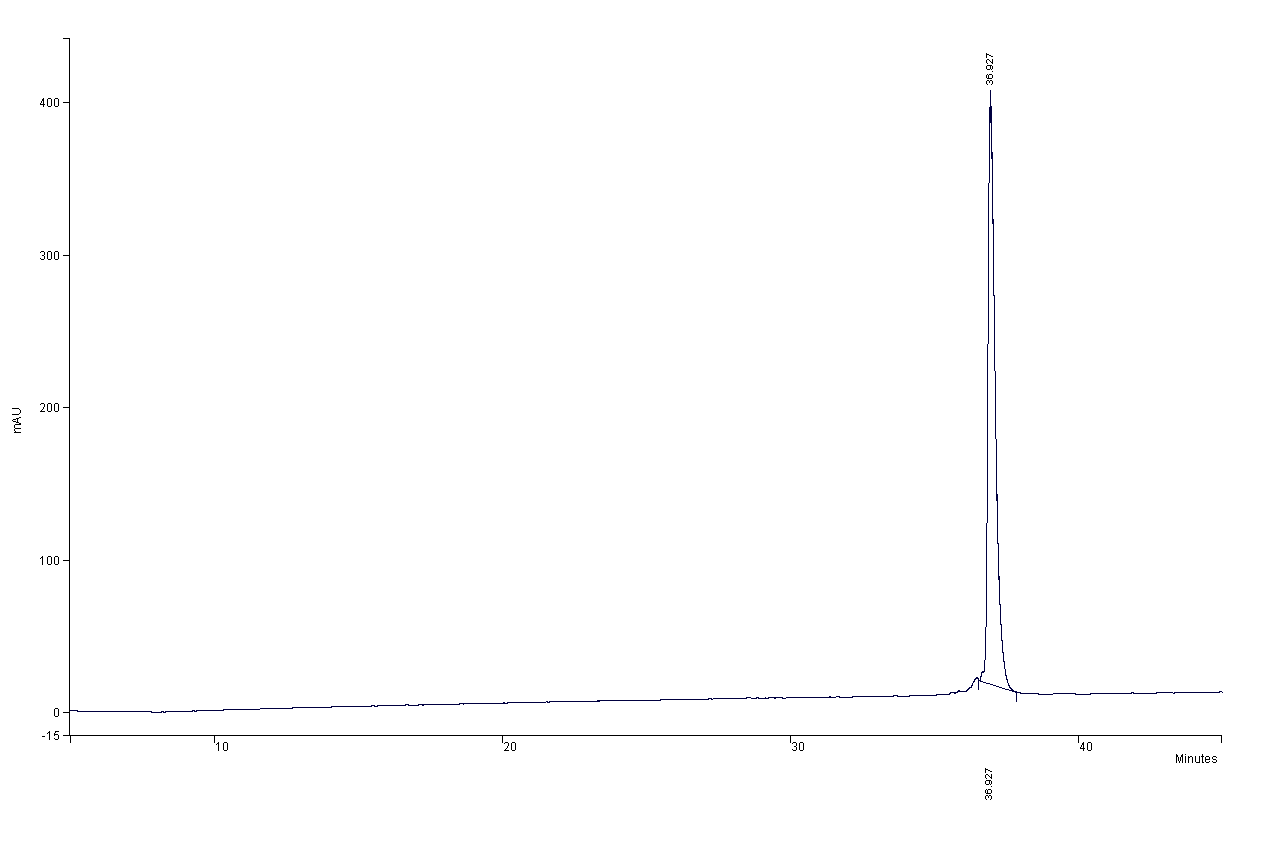  **A** | 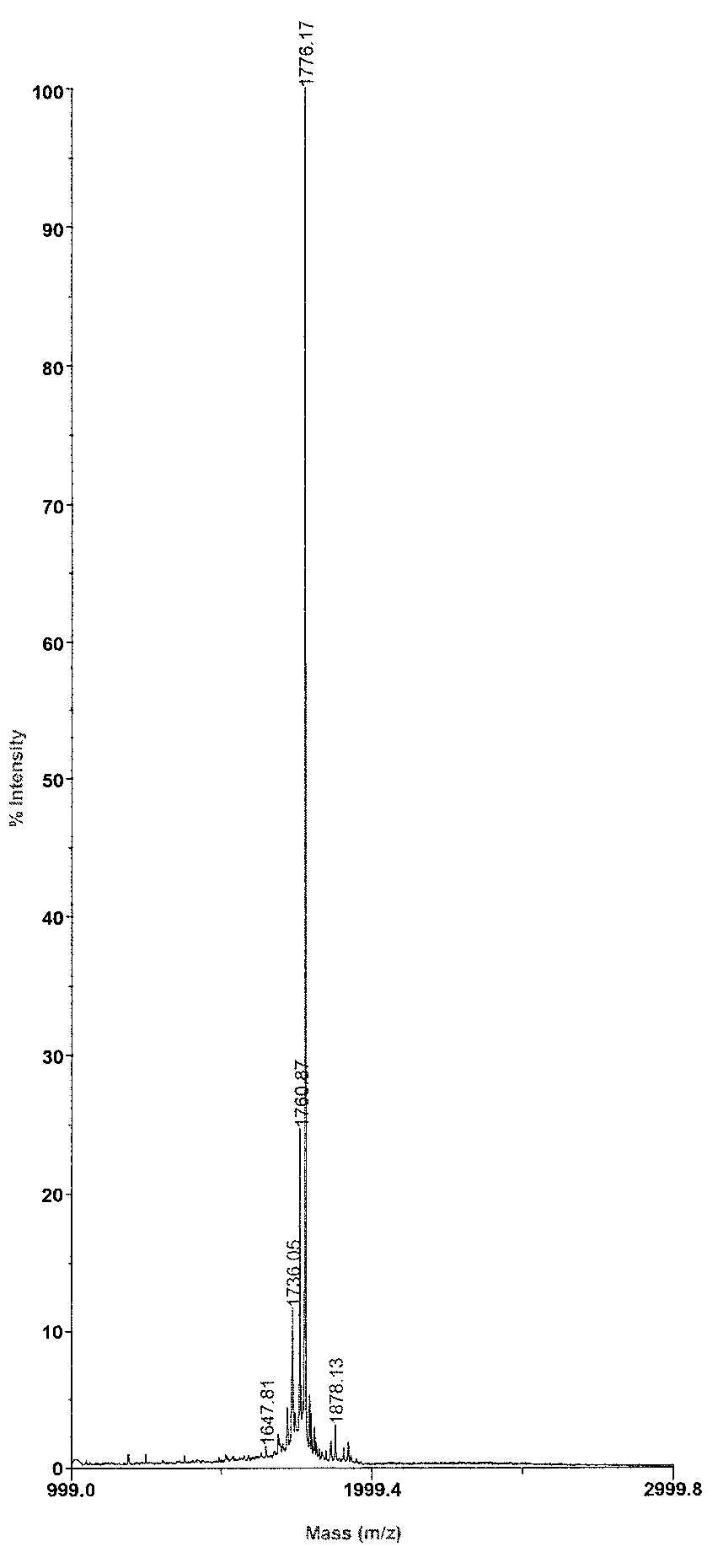 |
| --- | --- |

| 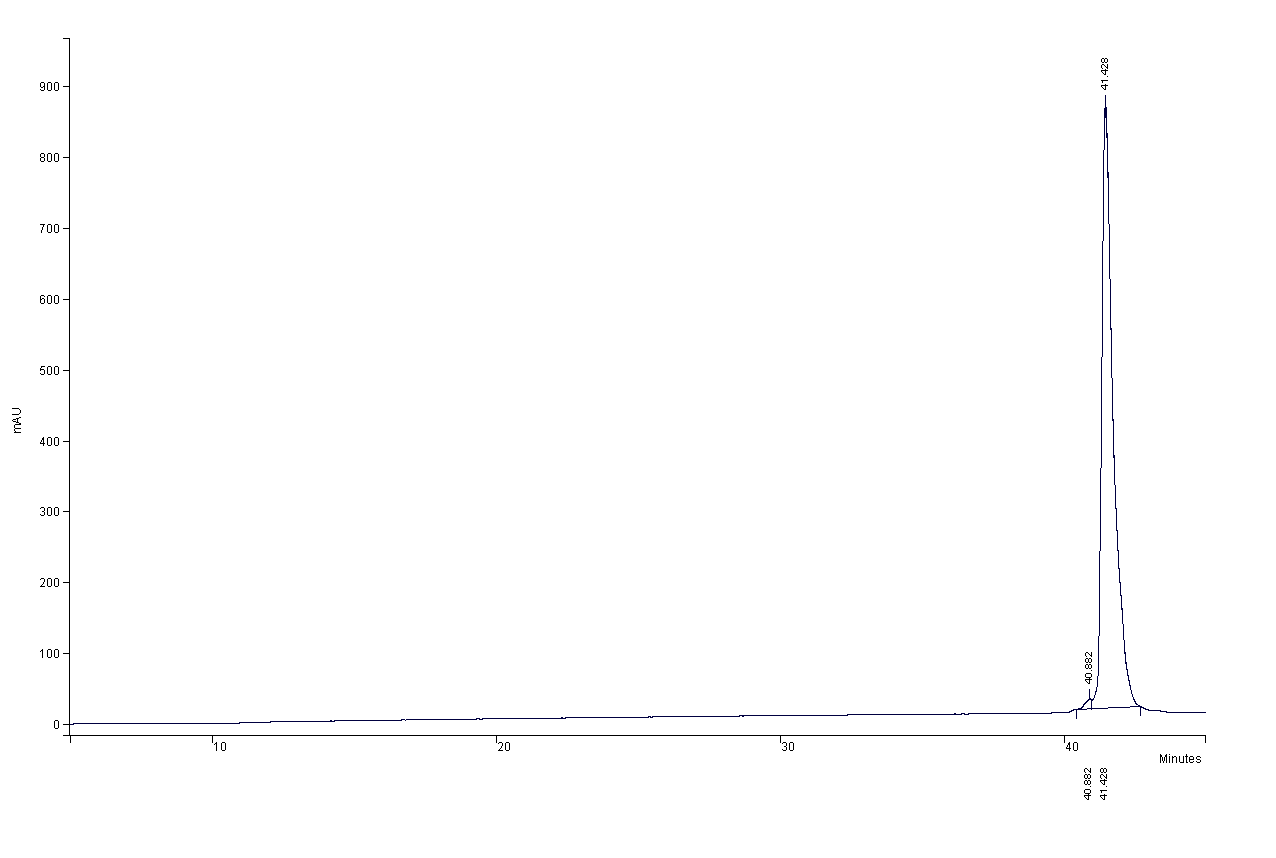  **B** | 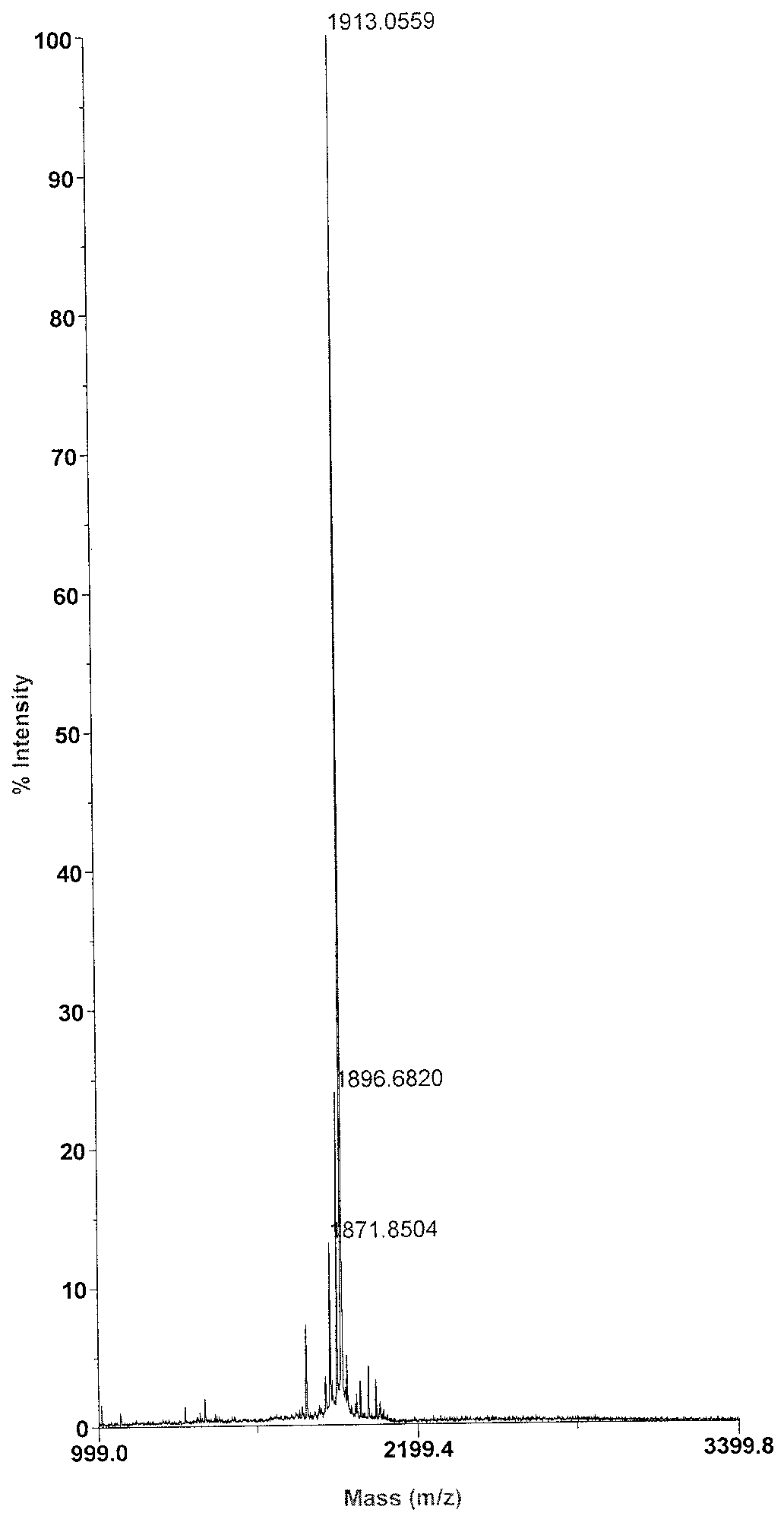 |
| --- | --- |

Supplement: Figure S1 — Two examples of analytical RP-HPLC profiles and corresponding MS spectra obtained for OxP peptides. Panel A - OxP-5; Panel B - OxP-11D. (0.13 MB DOC) [file pone.0010181.s002.doc]
